# Characterizing the Performance of Gas-Permeable Membranes as an Ammonia Recovery Strategy from Anaerobically Digested Dairy Manure

**DOI:** 10.3390/membranes7040059

**Published:** 2017-10-07

**Authors:** Melanie Fillingham, Andrew C. VanderZaag, Jessica Singh, Stephen Burtt, Anna Crolla, Chris Kinsley, J. Douglas MacDonald

**Affiliations:** 1 Agriculture and Agri-Food Canada, Ottawa Research and Development Centre, Ottawa, ON K1Y 4X2, Canada; melaniefillingham@gmail.com (M.F.); jess_singh_@hotmail.com (J.S.); Stephen.burtt@agr.gc.ca (S.B.); 2 Campus d’Alfred, University of Guelph, Alfred, ON N1G 2W1, Canada; anna.crolla@ontario.ca (A.C.); ckinsley@uottawa.ca (C.K.); 3 Environment Canada, Pollutant Inventory and Reporting Division, Gatineau, QC J8X 4C6, Canada; Douglas.MacDonald2@canada.ca

**Keywords:** ammonia recovery, air pollution, dairy manure emission mitigation, gas-permeable membranes, ammonia emissions, biogas, ammonia inhibition

## Abstract

Capturing ammonia from anaerobically digested manure could simultaneously decrease the adverse effects of ammonia inhibition on biogas production, reduce reactive nitrogen (N) loss to the environment, and produce mineral N fertilizer as a by-product. In this study, gas permeable membranes (GPM) were used to capture ammonia from dairy manure and digestate by the diffusion of gaseous ammonia across the membrane where ammonia is captured by diluted acid, forming an aqueous ammonium salt. A lab-scale prototype using tubular expanded polytetrafluoroethylene (ePTFE) GPM was used to (1) characterize the effect of total ammonium nitrogen (TAN) concentration, temperature, and pH on the ammonia capture rate using GPM, and (2) to evaluate the performance of a GPM system in conditions similar to a mesophilic anaerobic digester. The GPM captured ammonia at a rate between 2.2 and 6.3% of gaseous ammonia in the donor solution per day. Capture rate was faster in anaerobic digestate than raw manure. The ammonia capture rate could be predicted using non-linear regression based on the factors of total ammonium nitrogen concentration, temperature, and pH. This use of membranes shows promise in reducing the deleterious impacts of ammonia on both the efficiency of biogas production and the release of reactive N to the environment.

## 1. Introduction

Ammonia (NH_3_) emissions from livestock manure are an important source of reactive nitrogen (N) release to the environment, impacting the health of humans and livestock, and resulting in N loss from the farm production cycle negatively affecting profitability. Loss of ammonia decreases the fertilizer value of digestate. Nitrogen fertilizers are then required for crop production, contributing to additional ammonia emissions and adding an economic burden to farmers. Ammonia emissions contribute to eutrophication of surface water bodies, soil acidification, and particulate matter formation [1]. Ammonia is a precursor to PM_2.5_ (particulate matter with an aerodynamic diameter of 2.5 µm or less), which has negative human health impacts [2]. At the same time, ammonia has a harmful impact on manure treatment processes, such as anaerobic digestion (AD). The AD process digests organic matter to produce renewable energy from the captured methane but in the process, mineralizes nitrogen to ammoniacal form. At high concentrations ammonia can inhibit the methane formation process during AD [3]. Managing ammonia in manure can result in increased efficiencies in terms of farm productivity and improved efficiency in manure treatment through AD. Moreover, there is a potential win-win if captured ammonia is in a liquid form that could be used as fertilizer for crops, offsetting synthetic fertilizer.

**Figure 1:**
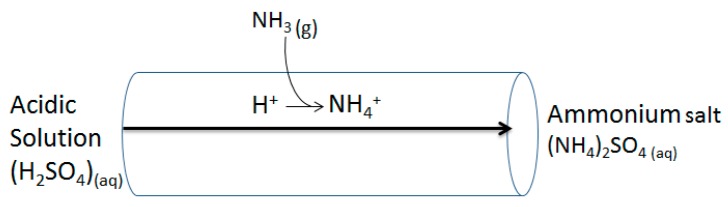
Schematic demonstrating the diffusion of ammonia gas (NH_3_) across the gas-permeable membrane and the reaction with the hydrogen ion (H^+^) donated from the acid to form liquid ammonium (NH_4_) which is captured.

Techniques and technologies to capture ammonia have been developed in attempts to reduce these negative effects in biodigesters, such as acid spray scrubbers, biofilters, acidified clays, and addition of sodium hydrogen sulphate [4]. Polytetrafluoroethylene (ePTFE) gas permeable membranes (GPM) filled with dilute acids have been successful in capturing ammonia gas from liquid manures (LM) [5], but have not yet been tested in dairy manure digestate. The ePTFE tubing has microscopic pores and is hydrophobic, allowing only gaseous diffusion. Mass transfer occurs across the membrane when there is a concentration difference, following Fick’s law of diffusion from high to low concentration. Ammonia gas diffuses through the GPM from the LM side into the sulphuric acid side, where it reacts to form aqueous ammonium sulphate, a salt that is also a commercial fertilizer, shown by Equation (1) and Figure 1.

(1) $$2{\mathrm{NH}}_{3}+H_{2}{\mathrm{SO}}_{4}↔{\left({\mathrm{NH}}_{4}\right)}_{2}{\mathrm{SO}}_{4}$$

Multiple parameters influence the flux across the membrane and the capture rate of ammonia, such as, the surface area of the GPM, the flow rate of the acid throughout the GPM, the gaseous ammonia concentration in LM, and the pH of the recipient acid [5,6]. Increasing the flow rate of sulphuric acid throughout the GPM has shown to increase the ammonia capture concentration in the acid solution, with an approximately 30% increase from 5.6 mL·min^−1^ to 36 mL·min^−1^ [5]. Mukhtar et al. [7] tested the surface area ratio of GPM to LM and found the optimal ratio to be 1:3.25 to achieve 50% ammonia extraction in less than 20 days. Over time, the pH of the acid solution increases as ammonium salt is formed, while the pH of the LM decreases [7]. Maintaining the recipient acid at pH of 2 (with an automated dosing pump and concentrated acid) resulted in the greatest removal of ammonia when compared to pH of 3, 4 and 5 [5].

The concentration differential across the membrane is the driving force of diffusion. Gaseous ammonia concentration is a function of manure characteristics, especially pH, temperature, and TAN concentration, as described by the following equations. The chemical equilibrium between TAN species, ammonia (NH_3_) and ammonium (NH_4_^+^), in aqueous solutions is shown in Equation (2).

(2) $${\mathrm{NH}}_{4\left(\mathrm{aq}\right)}^{+}↔{\mathrm{NH}}_{3}+H^{+}$$

This equilibrium shifts towards the ammonia phase at higher pH and temperature. The concentration of unionized ammonia (NH_3_, right side of Equation (2) is referred to as the concentration of free ammonia nitrogen (FA-N, in mg·L^−1^), which can be calculated using Equation (3).

(3) $$\mathrm{FA}‒N=\left[\mathrm{TAN}\right]\;\times \frac{10^{\mathrm{pH}}}{e^{\frac{6344}{273+T}}+10^{\mathrm{pH}}}$$

where *T* is the temperature (°C), and [TAN] is the total ammonia nitrogen concentration (NH_3_ and NH_4_^+^, mg·L^−^^1^), and pH is the potential of hydrogen [8]. Due to the low pH on the acid side of the GPM, ammonia is only present on the LM side and the flux is always directed away from the LM and towards the acid. Ammonia gas and aqueous ammonia within a solution are in equilibrium, as demonstrated by Equation (4a), with the ratio described by Henry’s Law Constant, *K*_H_, as follows in Equation (4b).

(4a) $${\mathrm{NH}}_{3\left(g\right)}↔{\mathrm{NH}}_{3\left(\mathrm{aq}\right)}$$

(4b) $$K_{H}=\frac{{\mathrm{NH}}_{3\left(g\right)}}{{\mathrm{NH}}_{3\left(\mathrm{aq}\right)}}$$

The ratio is dependent on temperature and can be calculated using Equation (5), where *T* is temperature (K) [9]:

(5) $$K_{H}=1384+1.053^{293-T}$$

In the context of extraction of NH_3_-N from LM, a higher pH corresponds to a higher FA-N value (Equation (3)), which causes a higher concentration gradient across the membrane. With higher pH in the LM, the removal rate of ammonia increases [6]. Temperature plays a key role in the activity and concentration of the dissolved and aqueous ammonia, and in the partitioning between ammonia gas and aqueous ammonia (Equation (5)). Higher temperature will result in an equilibrium shift towards higher concentration of gaseous ammonia; however, Henry’s Law constant decreases, and therefore, the ratio of gaseous to aqueous ammonia is lower. Temperature has a more significant impact on the equilibrium shift towards the quantity of ammonia present than on Henry’s law constant, therefore, increasing the temperature will theoretically always increase capture. Transfer rates across the membrane are regulated by the concentration of gaseous free NH_3_.

Controlling ammonia within a digester improves methane production [3]. The digestion process in an anaerobic digester is exothermic and the temperature ranges from 15 °C to 60 °C, depending on reactor type [10]. The process can also increase the pH of manure to 8 or higher (due to bicarbonate formation during fermentation). Ammonia is excreted by livestock and also results from the digestion of proteins, urea, and nucleic acids in manure [11]. Concentrations of mineral N in the ammoniacal form reported by Brown [12] were 2645, 1601 and 5567 ppm for hog, dairy and poultry manures, respectively (with substantial variability associated with dry matter (DM) content). These conditions (high temperature, pH and TAN concentration) within an AD are optimal for ammonia capture. Depending on the type of reactor and the operating conditions, the TAN inhibition occurs from 4000–10,000 mg·L^−1^ [3]. Avoiding these conditions will improve reactor efficiency. Furthermore, when NH_3_ concentrations are unchecked, digestate storage leads to high ammonia losses [13], therefore, capturing ammonia also increases the environmental benefit of AD.

The objectives of this research were to test ePTFE GPM to (1) determine the optimal capture rate conditions considering total ammonium nitrogen (TAN) concentration, temperature, and pH, and (2) to mimic the conditions within a dairy manure anaerobic digester and in so doing, to evaluate whether it could be feasible to adopt within the AD reactor. If successful, GPMs could be implemented into the anaerobic digester cycle, whether within or prior, to reduce the ammonia inhibition of methanogenesis. Research has shown success in the use of GPM for ammonia recovery of anaerobically digested swine manure, but there is a lack of research using dairy manure digestate [14]. It is hypothesized that with higher pH, temperature, and initial TAN concentration, there will be more capture due to more gaseous ammonia present, and that the conditions within an AD will increase ammonia capture compared to raw manure.

## 2. Results and Discussion

### 2.1. Time Series of Ammonia Capture

**Figure 2:**
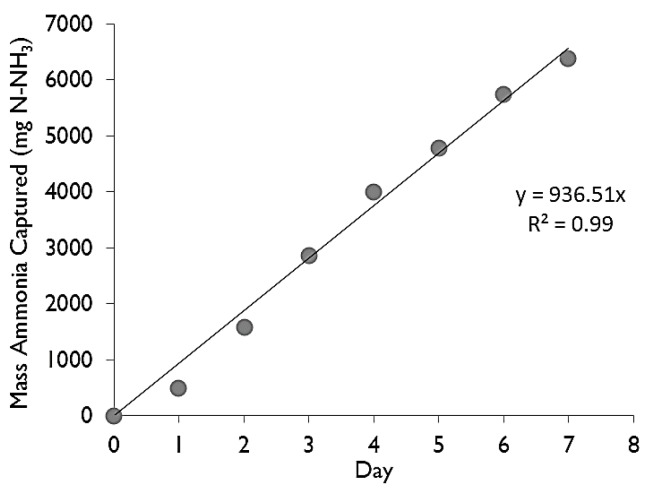
Representative time series of NH_3_ capture within the sulfuric acid (receptor) per day. Results from digestate (donor) with an initial TAN concentration of 1860 mg·L^−1^, initial pH of 7.7, and an initial temperature of 23.7 °C. The slope is taken as the capture rate (937 mg NH_3_·day^−1^).

Ammonia capture increased in a linear manner with time over the 7-day extraction period for all donor solutions (example shown in Figure 2). Capture rates (mg NH_3_·day^−1^) were calculated based on the slope of the linear regression. Trials involving ammonium chloride were manipulated so that the NH_3_ concentration in the donor solution was brought back up to the initial concentration each day. Trials involving raw manure (RM) and digestate (DG) were not manipulated in this way, therefore, the concentration of NH_3_ in the donor decreased with time. If ammonia is captured from a batch loaded reactor, it is expected that the capture rate would diminish with time. On the other hand, if ammonia is captured from a continuously fed reactor (such as a continuously fed and continuously mixed anaerobic digester) it is expected that the capture rate would remain at a steady-state.

### 2.2. Ammonium Chloride Solutions

In initial trials, ammonium chloride was used instead of liquid manure or digestate to allow for easy manipulations to parameters, such as the pH and TAN concentration.

#### 2.2.1. Effects of pH

**Figure 3:**
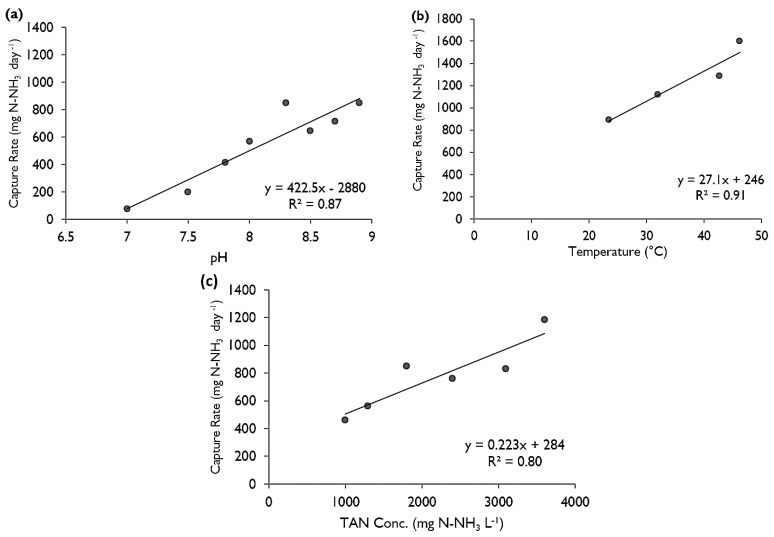
Comparison of the trapping rates achieved by altering (**a**) the pH, (**b**) the temperature, and (**c**) the concentration of ammonium chloride solution. The baseline parameters, i.e., those unchanged, were room temperature, 21–24 °C, 1800 mg N-NH_3_ L^−1^, and pH 8.3. Flux can be determined by dividing the daily capture rate by the surface area of the gas permeable membranes (GPM). All three regressions are statistically significant, *p* < 0.05.

The effects of pH were tested by performing multiple experiments in which the pH of the ammonium chloride changed (eight pH values from 7 to 8.9 were tested) but the concentration and temperature stayed consistent (at a target 1800 mg L^−^^1^ N-NH_3_ and room temperature, 22–24 °C). As expected, the rate of capture increased in a linear manner as the pH increased (*R*^2^ of 0.87, Figure 3a).

Over time, the pH of the ammonium chloride solution decreased as ammonia was removed. It was expected that at high pH, the capture rate would be greater, therefore, the pH was increased daily to the initial pH. In 24 h periods, the pH decreased and as a consequence daily trapping rates were the average rate measured over the 24 h, but the instantaneous trapping rates would decrease through each 24 h period. If a pH dosing device, similar to the one used for the recipient acid, was used to dose the ammonium chloride with concentrated sodium hydroxide when it dropped below a certain pH, the trapping rate would be further increased.

#### 2.2.2. Effects of Temperature

Increasing the temperature increases the total concentration of free ammonia (NH_3(g)_ and NH_3(aq)_) present in the solution, as indicated by Equation (3). However, increasing the temperature also decreases the Henry’s law constant, which is the ratio of gaseous ammonia to aqueous ammonia (Equations (4) and (5)—it is the gaseous ammonia that is captured by the use of GPM. Although the ratio of gaseous to aqueous ammonia is less at higher temperatures, the increase in free ammonia due to the higher temperature still allows for a greater concentration of gaseous ammonia when compared to a similar scenario at a lower temperature.

Temperature had a positive effect on the capture rate, with higher temperatures having higher fluxes (Figure 3b). The relationship was highly linear (*R*^2^ = 0.91). The temperature was controlled to remain constant throughout the experiments. The TAN concentration of the ammonium chloride was difficult to keep at the target value in the heated experiments as the higher temperatures increased evaporation and volatilization.

#### 2.2.3. Effects of Concentration

Different concentrations of TAN (1000 mg·L^−^^1^, 1300 mg·L^−^^1^, 1800 mg·L^−^^1^, 2400 mg·L^−^^1^, 3100 mg·L^−^^1^ and 3600 mg·L^−^^1^ N-NH_3_) were tested while holding pH at 8.3 and maintaining room temperature. Increasing the concentration of the TAN in the ammonium chloride solution resulted in a linear increase in the trapping rate of ammonia (*R*^2^ = 0.80) (Figure 3c). The decrease in TAN concentration in the donor solution was not as important as the decrease in pH, therefore, the effect it would have over the trapping rate through the 24 h was likely minimal. Overall, there was 4–6% decrease in TAN daily.

#### 2.2.4. Combined Effects on Capture Rate in Ammonium Chloride Solution

**Figure 4:**
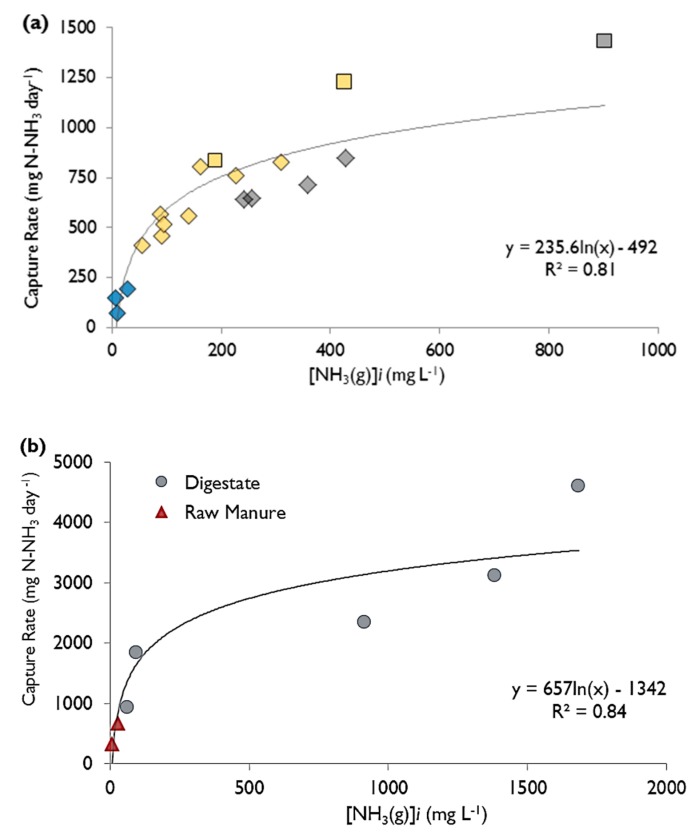
The capture rate of ammonia versus initial concentration of gaseous ammonia within the donor solution of (**a**) ammonium chloride, where experimental conditions varied the concentration of TAN, pH and temperature. Symbol type indicates temperature range (♦: 22 to 25 °C, □: ≥30 °C) and symbol colour indicates pH range (blue: pH 7.0 to 7.5; yellow: pH > 7.5 to 8.3; gray: pH > 8.3); (**b**) digestate (●) or raw manure (▲). For the experimental parameters corresponding to each data-point in (**b**), refer to Table 1.

Combining results from all experiments conducted with ammonium chloride, the NH_3_ capture rates corresponded to the gaseous ammonia concentration and fit to a logarithmic regression (*R*^2^ = 0.81) (Figure 4a):

$$\mathrm{CR}=235.6\times \ln\left[{\mathrm{NH}}_{3\left(g\right)}\right]-492.2$$

where CR is the capture rate of ammonia, in mg N-NH_3_ day^−^^1^, and [NH_3(g)_] is the concentration of gaseous ammonia, in mg·L^−^^1^, calculated using Equations (3)–(6). The relationship shows a rapid increase of capture rate when the concentration of ammonia gas increases between 0 and 50 mg·L^−^^1^, and a less rapid increase of capture rate with increase of concentrations of ammonia gas above 500 mg·L^−^^1^. The trend is almost linear when below 200 mg·L^−^^1^.

The flux values—calculated by dividing the daily capture rate by the GPM surface area—were positive for every trial and ranged from less than 1 g·m^−^^2^·day^−^^1^ (when the pH was 7 and the concentration of ammonia gas in solution was very low) up to 20 g·m^−^^2^·day^−^^1^ (when the pH and temperatures were higher). Changes in pH of the ammonia chloride had the greatest effect on the overall results, as expected by the relationship in Equation (3) followed by temperature and TAN concentration.

### 2.3. Liquid Manure and Digestate Results

When the digestate and raw manure trials were conducted (Table 1), the trapping rates (Figure 4b) were higher than expected following the trend formed by using ammonium chloride, for the corresponding gaseous ammonia concentrations (Figure 4a). The relationship between gaseous ammonia and the capture rate was logarithmic (*R*^2^ = 0.84) when raw manure and digestate were tested:

$$\mathrm{CR}=656.9\ln\left[{\mathrm{NH}}_{3\left(g\right)}\right]-1341.8$$

where CR is the capture rate of ammonia, in mg N-NH_3_ L^−^^1^·day^−^^1^ and [NH_3(g)_] is the concentration of gaseous ammonia, in mg·L^−^^1^, calculated using Equations (3)–(6).

Digestate pH fluctuated throughout the experiment perhaps due to reduced buffering capacity compared to raw manure. Raw manure pH steadily decreased throughout each trial. The recipient solution remained transparent and colourless, indicating that no solids passed through. The first few days of the digestate experiments, there were obvious bubbles (likely CO_2_ and CH_4_) within the recipient solution that fizzed when stirred. The bubbles only occurred at the start of the experiment. The raw manure recipient solution did not show any sign of the bubbles. The bubbles were present in the digestate obtained from both farms. Other volatile gases may have been captured in the first few days that later were lost from solution.

Digestate and manure have a much higher solids content than the ammonium chloride solution. The solids retained gas bubbles in the liquid. Bubbles could be seen on the walls of the chamber and could be the reason that higher capture was occurring; the gaseous ammonia was staying in solution instead of evaporating. These pockets of gas could also explain why there was a greater increase in volume captured for the heated digestate samples when compared to heated ammonium chloride samples; water vapour was staying beneath the liquid. Ahn et al. [6] studied solids concentration versus the capture rate within a pressure vessel system, reporting that higher solids content decreased capture rate.

The increasing trend in the daily captured ammonia for the raw manure and digestate samples tended to be linear, although pH was not controlled. It is predicted that if the trial periods were longer, the rate of ammonia capture would level off. The GPM used for the digestate samples rinsed clean in colour after the trial was over. The GPM used for the raw manure was stained after the trial and the colour could not be physically removed. Since the rate of capture did not change between the seven days of the trial, it is assumed that staining did not impact the capture rate and did not block the GPM pores.

Characteristics of digestate (high TAN and high pH) made it a more favourable material for ammonia capture than raw manure. Digestate had substantially higher gaseous ammonia concentrations than raw manure. It demonstrated a substantially higher ammonia capture rate, with the maximum being 4600 mg·day^−^^1^ (when gaseous ammonia was 1684 mg·L^−^^1^) compared to a maximum capture rate using raw manure of 683 mg·day^−^^1^ (when gaseous ammonia was 27.2 mg·L^−^^1^). The daily percent mass captured of ammonia from digestate was 12.8%, whereas raw manure was only 2.9% (Table 1). The total mass (over 7 days) of captured ammonia within digestate was as high as 12,900 mg N-NH_3_, whereas raw manure only reached 3540 mg N-NH_3_ in the same time period (per 1 L receptor acid). The daily mass reduction (from the RM and DG) indicate a better removal from digestate than raw manure (Table 1). If no ammonia was being produced in the donor solution it would be expected that the mass reduction rate (from the donor solution) be equal to the mass capture rate (into the receptor); however, this was not the case as they were quite different at times (Table 1). In other words, the mass of ammonia captured was not equal to the difference between the initial and final mass in the digestate or raw manure because of organic N being mineralized to inorganic N throughout the trial.

### 2.4. Trapping Water Vapour

At room temperature, the membranes proved to be hydrophobic as the volume of recipient trapping solution remained constant. At increased temperature, however, the volume of the recipient solution increased by as much as 300 mL·day^−^^1^ above 40 °C. Increased evaporation within the chambers was apparent (condensation on the walls and lid), and water vapour was likely diffusing through the GPM, then condensing and increasing the volume of trapping solution. Trials running with digestate saw the greatest increase in recipient volume (300 mL·day^−^^1^) compared to ammonium chloride (100 mL·day^−^^1^). In trials where the recipient solution volume increased, the volume and concentration was taken into account when calculating trapping efficiency. Subsequently, tests were conducted using only water (instead of LM or ammonium chloride), and the results showed that the recipient solution increased in volume due to transfer of water vapour across the GPM. The recipient solution remained transparent and colourless even when liquid manure or digestate was used, indicating that there was no transfer of solids.

### 2.5. Future Research

Several areas for further study were identified:

- Determining ways to avoid transfer of water vapour when GPM is used at high temperature.
- Evaluating the benefit of NH_3_ removal using the GPM system on methane gas production in a pilot-scale biodigester.
- Evaluating options to increase the NH_3_ capture rate, e.g., more concentrated acid, acid with more available donor protons, higher flowrate of trapping solution.
- Evaluating the fertilizer value of the trapped nitrogen solution.
- Longer duration trials with digestate or RM.
- Evaluating clogging of the GPM with time.
- Use aeration to increase pH and recovery rate, such as done by Dube et al. [14].

Characterizing alternative species being captured and evaluating ways of improving membrane specificity (including the examining unintended water vapour capture).

## 3. Materials and Methods

### 3.1. Gas-Permeable Membrane

**Figure 5:**
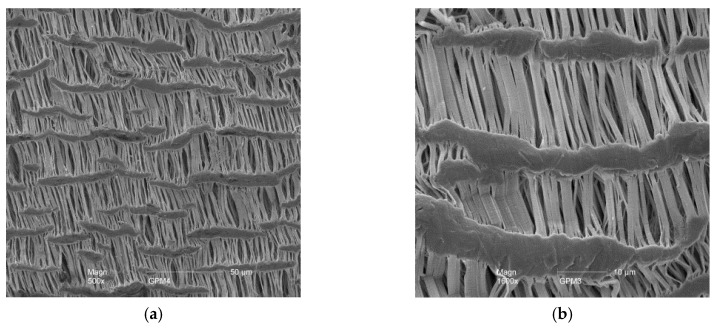
Microscopic photographs of the ePTFE membrane at 500× magnification (**a**) and 1600× magnification (**b**).

The gas permeable tubular membranes used were made from expanded polytetrafluoroethylene (ePTFE) (FL1001, Phillips Scientific, Mahwah, NJ, USA) (Table 2). Pore characteristics of the ePTFE were examined with an environmental scanning electron microscope (XL-30 ESEM, Philips Scientific, Mahwah, NJ, USA) (Figure 5).

### 3.2. System Configurations

**Figure 6:**
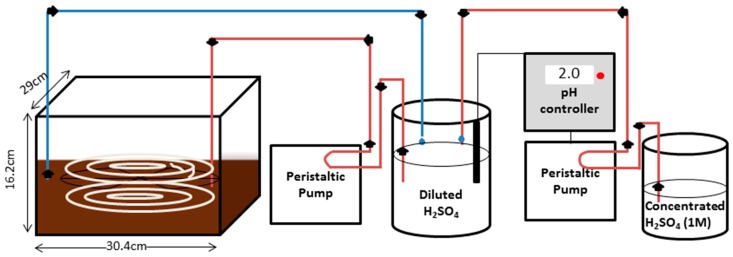
Schematic of ammonia capture system. Diluted H_2_SO_4_ is pumped from a 1 L beaker throughout the system, through the double-web structure GPM which is immersed in a donor solution (NH_4_Cl, digestate or manure) and back to the 1 L beaker. When the pH of the diluted H_2_SO_4_ exceeds 3, a controller activates a pump transferring 1M H_2_SO_4_ to the diluted H_2_SO_4_ beaker, lowering the pH.

The overall GPM system consisted of two identical 14 L chambers (30.4 cm width, 29 cm length and 16.2 cm height) made of 1/4 Plexiglas which were filled with either 8 L of ammonium chloride solution, 8 L digested dairy manure, or 8 L raw dairy manure (Figure 6). Aqueous ammonium chloride was used for practical laboratory testing [15]. The GPM was completely immersed in the liquid in a web-like configuration to optimize the contact area within the chamber. The web configuration was made using a stainless steel circular base with four crossed arms, dividing the circle into eight sections of equal size. The circular base had three levels of duplicate holes on each arm, one with a diameter of 26 cm, one with a diameter of 17.3 cm and one with a diameter of 8.7 cm, all from the same centre point. Cable clips were clipped into the holes, one on the top side of the pair and one on the bottom side. The GPM tubing was then strung through these clips in a spiral configuration. The length of the tubing was held constant at 360 cm for each trial, resulting in a GPM surface area of 905 cm^2^.

Tubing throughout the rest of the system was made of Tygon (OD 7 mm, ID 4 mm). Sulphuric acid (0.001 M, initial pH 2) was circulated at a flow rate of 18.2 mL·min^−1^ from a 1 L closed beaker (#2070350000, Fisher Scientific, Ottawa, ON, Canada) through the immersed GPM tubing and back to the 1 L beaker by means of a Gilson Minipuls 2 multichannel peristaltic pump. The closed beaker contained 800 mL of acid trapping solution, after the tubing was filled (1 L total being circulated). Industrial flat tip pH electrodes (#HI 1006-1007, Hanna Instruments, Laval, QC, Canada) were submerged in each of the 1 L beakers of diluted sulphuric acid. The pH electrodes were connected to controllers (BL 931700, Hanna Instruments, Laval, QC, Canada) that activated peristaltic pumps (Fisher Scientific, Ottawa, ON, Canada) to add concentrated 1M sulphuric acid from a 500 mL beaker when the pH within the 1 L beaker exceeded 3. The pH of the acid trapping solution within both 1 L beakers were logged every half hour using two data loggers (DataSuite MicroLite II LITE5032P-4/20-A, Fourier Technologies, Orlando Park, FL, USA).

Temperature and pH of the ammonium chloride solutions, digested manure, or raw manure, were measured and logged once every half hour by an IntelliCAL pH electrode with a built-in temperature sensor (PHC28101, Hach Company, Loveland, CO, USA) connected to a portable meter (HQ40d53000000, Hach Company, Loveland, CO, USA). The ammonium chloride solutions were brought back to the target pH daily by adding 1 M sodium hydroxide. The pH of manure and digestate was not altered.

Ammonia concentrations were tested daily using the HACH Meter attached with a HACH IntelliCAL ammonia ISE electrode (ISENH318103, Hach, Loveland, CO, USA). The TAN concentrations within the liquid manure were only measured at the start and finish of the trial. The TAN concentrations of the sulphuric acid trapping solution were tested daily in all experiments using the Hach electrode and confirmed by analysing a 15 mL subsample using Flow Injection Analysis [17]. Daily subsamples were refrigerated at 5 °C until analysis. The TAN concentrations in the ammonium chloride trials were brought back to the target concentration daily, sampling before and after concentration adjustment, to ensure similar conditions throughout the trial.

### 3.3. Experimental Approach

Eighteen trials were conducted to test the performance of the GPM system and to characterize the effects of changing the three parameters that affect the concentration of aqueous ammonia: temperature, concentration, and pH. Each trial lasted seven days. To represent the characteristics of different manures and digested manures, ammonium chloride solutions were prepared with specific levels of pH and TAN. Ammonium chloride was selected as a “synthetic manure” because it enabled consistency across trials which would not be possible with manure, and it facilitated manipulation of the desired characteristics which would be more difficult to alter using liquid manure. After trends and relationships among variables were observed using ammonium chloride, the system was switched over to using liquid dairy manure and digestate obtained from two commercial dairy farms, each with a biodigester receiving raw manure and co-substrates from other industries.

In the initial experiments, two parameters were held constant while the third was changed to analyse the influence of each separately. The “base conditions” were a pH of 8.3, an initial TAN concentration of 1800 mg N-NH_3_ L^−1^, and room temperature (21–24 °C). Previous studies have found that pH of the liquid manure (or ammonium chloride) decreased as ammonia was captured and the TAN concentration decreased [7]. Within a continuously mixed AD, manure would consistently be added and removed at steady state, therefore maintaining near constant concentration and pH. To mimic this, the pH and concentration of ammonium chloride were brought back up to the target each day at a consistent time, using the HACH pH and ammonia probes to determine the amount of NaOH and NH_4_Cl to be added to achieve base conditions.

The first parameter that was changed was pH. A trial was conducted representing “normal” manure conditions (i.e., pH 8, room temperature (21–24 °C), and 1800 mg N-NH_3_ L^−1^). While keeping temperature and concentration constant, multiple trials were performed to test pH values of 7, through 8.9.

The second parameter changed was temperature. The temperature was increased and maintained with custom-made, programmable heating pads below each chamber that evenly distributed heat over the base of the chamber and a thermistor within the chamber measured the temperature and adjusted the heat supplied. Data from the HACH pH and temperature probe were recorded every half hour, to ensure the temperature was maintained at the target. The pH and concentration for each of the temperature tests were held constant, at 8.3 and 1800 mg N-NH_3_ L^−1^ respectively. Temperatures tested were room temperature (22–24 °C), 32, 42.5 and 46.2 °C.

The final parameter tested was concentration. The concentration was increased by simply starting with a stronger ammonium chloride solution. The concentrations tested, while pH and temperature remained constant were 1000, 1400, 1800, 2400, 3200 and 3500 mg N-NH_3_ L^−1^.

After the above trials using ammonium chloride were conducted, the system was switched from ammonium chloride to liquid manure and digestate to determine whether the findings and trends were transferrable. The procedure changed such that the concentration and pH were left to decrease over the trial (not being adjusted daily) and the ammonia concentration was only tested at the start and end of the trial, while the concentration of ammonia captured was still measured daily. Raw manure was collected twice from the same farm in Eastern Ontario—in one case the characteristics were 1430 mg N-NH_3_ L^−1^, pH 7.0 and 8.8% dry matter (DM), and the other case had characteristics 3280 mg N-NH_3_ L^−1^, pH 7.2 and 9.2% DM. Digestate was collected from two commercial dairy farms within Ontario, one of which was the same location the raw manure was collected. The digestate was collected on two separate occasions from the first farm (same location as the RM), with the conditions being 1860 mg N-NH_3_ L^−1^ with pH 7.65 and 5.0% DM, and 1800 mg N-NH_3_ L^−1^ with pH 7.55 and 5.5% DM, respectively. Sufficient digestate was collected from the second farm on one occasion for multiple trials, with the conditions being 6130 mg N-NH_3_ L^−1^ with pH 8.5 and 5.2% DM. All manure/digestate was stored at 5°C when not in use. The temperature of the RM and digestate was first run at room temperature for all samples. The digestate was also tested at a temperature of 38 ± 1 °C for samples from both locations.

### 3.4. Data Analysis

The concentration of the recipient sulfuric acid solution was tested daily at approximately the same time of day. The concentration was converted into mass by multiplying by the volume of the recipient solution. The mass captured versus time (in days) was then plotted for each trial and a linear regression was performed to find the rate at which ammonia was being trapped, hereon termed as the “capture rate” (in mg·day^−1^). When two of the three parameters were held constant across trials, the third could be compared simply by the change in the trapping rate.

To compare trials of different pH, temperature and concentrations, the concentration of ammonia gas corresponding to the liquid manure/ammonium chloride solution was calculated using Equation (6), which was derived knowing that Henry’s Law constant (*K*_H_) is a ratio between NH_3(s)_ and NH_3(aq)_ (Equation (4b)) and that free Ammonia (FA) is the summation of NH_3(s)_ and NH_3(aq)_.

(6) $$\left[{\mathrm{NH}}_{3\left(g\right)}\right]=\frac{\mathrm{FA}}{\left(1+\frac{1}{K_{H}}\right)}$$

Equation (6) takes into consideration the three variables that affect the concentration and hence the mass transfer across the GPM: temperature, pH and TAN concentration. The ammonia gas concentration for each trial was then calculated and used in regression analysis with the trapping rate. The concentration of ammonia gas can be found for both the LM/ammonium chloride side of the GPM or for the sulphuric acid recipient side. The capture rate (mg·day^−1^) was calculated from a linear regression fitted to the trend from the mass captured vs. time (an example can be seen in Figure 1). Flux (*J*) was determined from the mass captured per day divided by the surface area of the GPM tubing, as shown in Equation (7).

(7) $$J=\frac{\mathrm{CR}}{A}$$

where CR is the capture rate (mg·day^−^^1^) of ammonia gas in the trapping solution (sulfuric acid), and *A* is the area (m^2^) of the gas permeable membrane.

The flux across the membrane can also be described following the mass transfer model expressed in Equation (8) [5,9]:

(8) $$J=K_{m}\left(C_{1}-C_{2}\right)$$

where *J* is the mass flux per area (g·m^−^^2^·s^−^^1^), *K*_m_ is the mass transfer coefficient, and *C*_1_ and *C*_2_ are the concentrations of the ammonia gas (i.e., [NH_3(g)_] from Equation (6)) in the donor solution (LM or ammonium chloride) and the trapping solution (sulfuric acid), respectively. The difference between *C*_1_ and *C*_2_, the concentration gradient, is the driving force of the flux according to Fick’s Law. The mass transfer coefficient, *K*_m_, is dependent on characteristics of the gas permeable membrane, such as porosity and pore size, as well as on the flowrate of dilute acid within the membrane [6,16].

## 4. Conclusions

The use of ePTFE GPM was successful at reducing the ammonia concentration within liquid raw dairy manure and digestate and capturing it as ammonium sulphate, a valuable fertilizer. This is a low energy approach to isolating mineral nitrogen, as the only energy requirement is the pump. Relationships were developed between the ammonia capture rate and factors that affect the gaseous ammonia concentration (pH, TAN concentration, and temperature). As pH, TAN concentration or temperature increased, the capture rate increased. The liquid digestate and manure showed similar trends as the ammonium chloride but with higher capture rates. The natural conditions (higher pH and TAN concentration) of digested dairy manure make it more favourable for the use of ePTFE GPM to capture ammonia than raw manure. Over 1 week, recipient solutions reached concentrations as high as 12,900 mg N-NH_3_ L^−1^ from digestate_,_ whereas raw manure reached concentrations of 3540 mg N-NH_3_ L^−1^ in the same time period. The daily capture of ammonia using digestate mimicking the conditions within an anaerobic digester was 12.8%. If this process could be done on a large scale, the solution created through this process could be applied to crops as fertilizer. This promises a win-win situation where a useful by-product is produced and simultaneously improves the efficiency of renewable energy production through anaerobic digestion. Digestate had a maximum capture rate of 4600 mg·day^−1^ whereas raw manure had a maximum capture rate of 680 mg·day^−1^, demonstrating that digestate is a more optimal material than manure for ammonia capture.

## Figures and Tables

**Table 1:** Summary of results and donor characteristics from trials using raw manure (RM) and digestate (DG). Each trial lasted 7 days.

Type	[TAN]_i_ mg·L^−1^	[TAN]_f_ mg·L^−1^	[NH_3(g)_]_i_ mg·L^−1^	pH Initial	pH Final	*T* (°C)	Daily Mass Reduction ** (% TAN_i_ Day^−1^)	Daily Mass Captured ** (% TAN_i_ Day^−1^)	Capture Rate mg NH_3_-N Day^−1^	Flux g·m^−2^ ·Day^−1^
RM	1430	1210	7.5	7.4	6.7	23.9	2.2	2.9	331	3.7
RM	3280	2740	27.2	7.4	7.2	24.0	2.4	2.6	683	7.6
DG	1860	1050	59.6	7.7	7.7	23.9	6.2	6.3	937	10.4
DG	1800	1000	93.8	7.6	7.5	38.0	6.3	12.8	1844	20.4
DG	6130	5030	912	8.5	7.9	24.0	2.5	4.8	2353	26.0
DG	6130	3984	1380	8.5	7.8	33.0	5.0	6.3	3114	34.4
DG	6130	3981	1684	8.5	8.4 *	37.0	5.1	9.4	4611	51.0

* pH dropped to 7.76 then increased again after day 5. ** Mass Reduction (MR) reflects the change in TAN concentration in the donor solution (final–initial), while Mass Capture (MC) reflects the amount of TAN captured in the recipient solution relative to the initial TAN concentration in the donor solution (captured/initial). MR% is not equal to MC% because of production of TAN occurring in the donor solution from mineralized organic N concurrent with TAN removal.

**Table 2:** Physical properties of the Polytetrafluoroethylene (ePTFE) gas permeable membrane used in the study. Length was measured, surface area was calculated, and all others were provided by the manufacturer.

Length (cm)	Surface Area (cm^2^)	Inside Diameter (mm)	Outside Diameter (mm)	Porosity (%)	Mean Pore Diameter (µm)
360	904.8	6.72	8	83	2.4 ± 0.142
